# Functional Surfactants for Molecular Fishing, Capsule Creation, and Single-Cell Gene Expression

**DOI:** 10.1007/s40820-021-00663-x

**Published:** 2021-06-19

**Authors:** Mohammad Suman Chowdhury, Xingcai Zhang, Leila Amini, Pradip Dey, Abhishek Kumar Singh, Abbas Faghani, Michael Schmueck-Henneresse, Rainer Haag

**Affiliations:** 1 Institut Für Chemie Und Biochemie, Freie Universität Berlin, Takustrasse 3, 14195 Berlin, Germany; 2 John A. Paulson School of Engineering and Applied Sciences, Harvard University, Cambridge, MA 02138 USA; 3 School of Engineering, Massachusetts Institute of Technology, Cambridge, MA 02139 USA; 4 Berlin Institute of Health – Center for Regenerative Therapies, Charité Universitätsmedizin Berlin – CVK, Föhrer Str. 15, 13353 Berlin, Germany; 5 Berlin Center for Advanced Therapies, Charité Universitätsmedizin Berlin – CVK, Föhrer Str. 15, 13353 Berlin, Germany

**Keywords:** Dendronized fluorosurfactants, Droplet microfluidics, Microcapsules, Oxidation-responsive fluorosurfactants, Cell encapsulation

## Abstract

Creating a single surfactant that is open to manipulation, while maintaining its surface activity, robustness, and compatibility, to expand the landscape of surfactant-dependent assays is extremely challenging. We report an oxidation-responsive precursor with thioethers and multiple 1,2-diols for creating a variety of functional surfactants from one parent surfactant. Using these multifunctional surfactants, we stabilize microfluidics-generated aqueous droplets. The droplets encapsulate different components and immerse in a bioinert oil with distinct interfaces where an azide-bearing surfactant allow fishing of biomolecules from the droplets, aldehyde-bearing surfactant allow fabrication of microcapsules, and hydroxyl-bearing surfactants, with/without oxidized thioethers, allow monitoring of single-cell gene expression. Creating multifunctional surfactants poses opportunities for broad applications, including adsorption, bioanalytics, catalysis, formulations, coatings, and programmable subset of emulsions.

**Supplementary Information:**

The online version contains supplementary material available at 10.1007/s40820-021-00663-x.

## Highlights

- We have demonstrated how to create a parent surfactant that is open to manipulation, while maintaining its surface activity, robustness, and compatibility, to expand the landscape of surfactant-dependent microfluidic-droplet-based assays.
- To create a library of functional surfactants from the parent surfactant, an oxidation-responsive small building block with thioethers and multiple 1,2-diols was used as a head group, while a non-polar perfluoropolyether chain was used as a tail.

**Supplementary Information:**

The online version contains supplementary material available at 10.1007/s40820-021-00663-x.

## Graphical abstract

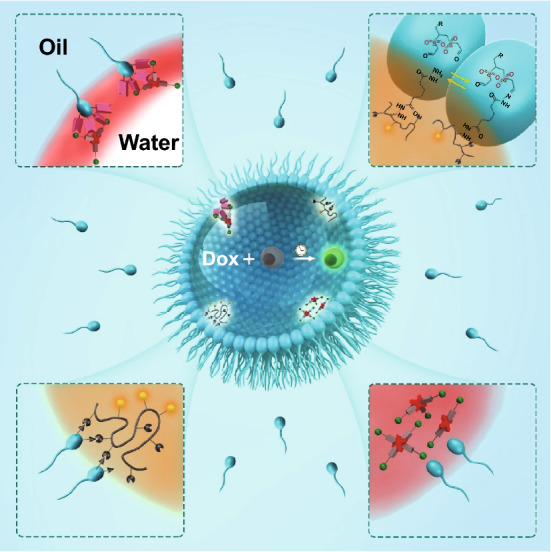

## Introduction

Surfactants play an inevitable role in modern science, including proteomics [1], nanotransporter design [2], and emulsions [3, 4]. They typically function to solubilize, contain, shield, and deliver contents of interest ranging from hydrophobic to hydrophilic, ionic to non-ionic, solid to liquid, and protein to single-cell. For example, fluorosurfactants have been widely used in poly(dimethyl siloxane) (PDMS)-based droplet microfluidics for many low-cost [5] but high-performance applications, such as single-cell barcoding [6], therapeutic antibody discovery [7], microcapsule fabrication [8], cell-mimetic compartment construction [9, 10], and digital enzyme-linked immunosorbent assay (ELISA) [11]. However, these surfactants including commercial ones cannot be used for multifunctional assays in drops as they lack functional groups and/or are vulnerable to manipulations [12–14]. They generally suffer from low valency, unsuitability for quantitative analysis, tedious customizability, and loss of robustness, efficiency, and surface activity after modification. Encapsulation, protection, and stabilization of drops, although possible by other elegant and powerful techniques and materials, such as nanoparticle jamming [15, 16], wrapping with/without prefabricated polymer films [17], and stimuli-responsive surfactants [18, 19], still lack compatibility with varying media, biological ingredients, and/or downstream manipulation, due to material types used, charge dependency, formulations conditions, and/or solvent incompatibility, which eliminate them from high-throughput applications.

Therefore, for advanced microfluidic applications, it is necessary to have fluorosurfactants whose functionality, valency, compatibility, and polarity can be readily customized on-demand. Customization of the functionality adds new chemical, biochemical, and/or biophysical properties, customization of the valency adds variable number and/or type of functional groups, and customization of the compatibility adds flexibility to work with elements, such as ionic and non-ionic molecules, biomolecules, and cells; and customization of the polarity adds freedom to choose a wide variety of polar and/or nonpolar molecules as functional groups for coupling with head groups. The possibility to customize all these will certainly leverage surfactants for many functional assays and enable creating a user-friendly subset of drop-based assays, which remained unnoticed and/or unsuccessful so far.

**Fig. 1:**
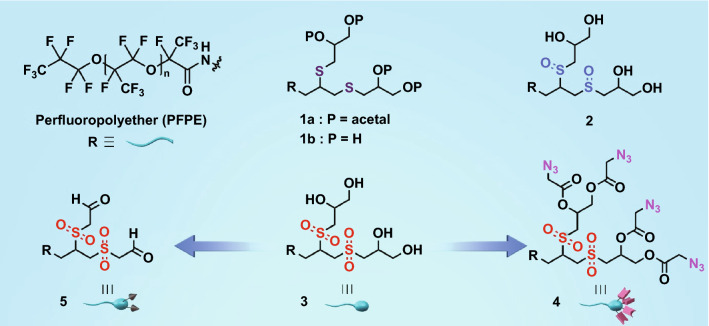
*Functional surfactants* The perfluoropolyether tail **R** is identical in surfactants **1a, 1b**, **2**, **3**, **4**, and **5**. Surfactant **1a** has multiple thioethers and the 1,2-diols are acetal protected (parent surfactant). Surfactants **1b**, **2**, and **3** contain multiple thioethers, sulfoxides, and sulfones, respectively, with four hydroxyl groups in each of them. Surfactant **3** was functionalized to create surfactant **4** with azido-moieties, while surfactant **5** with aldehyde groups was prepared by oxidizing the 1,2-diols in **3**

**Fig. 2:**
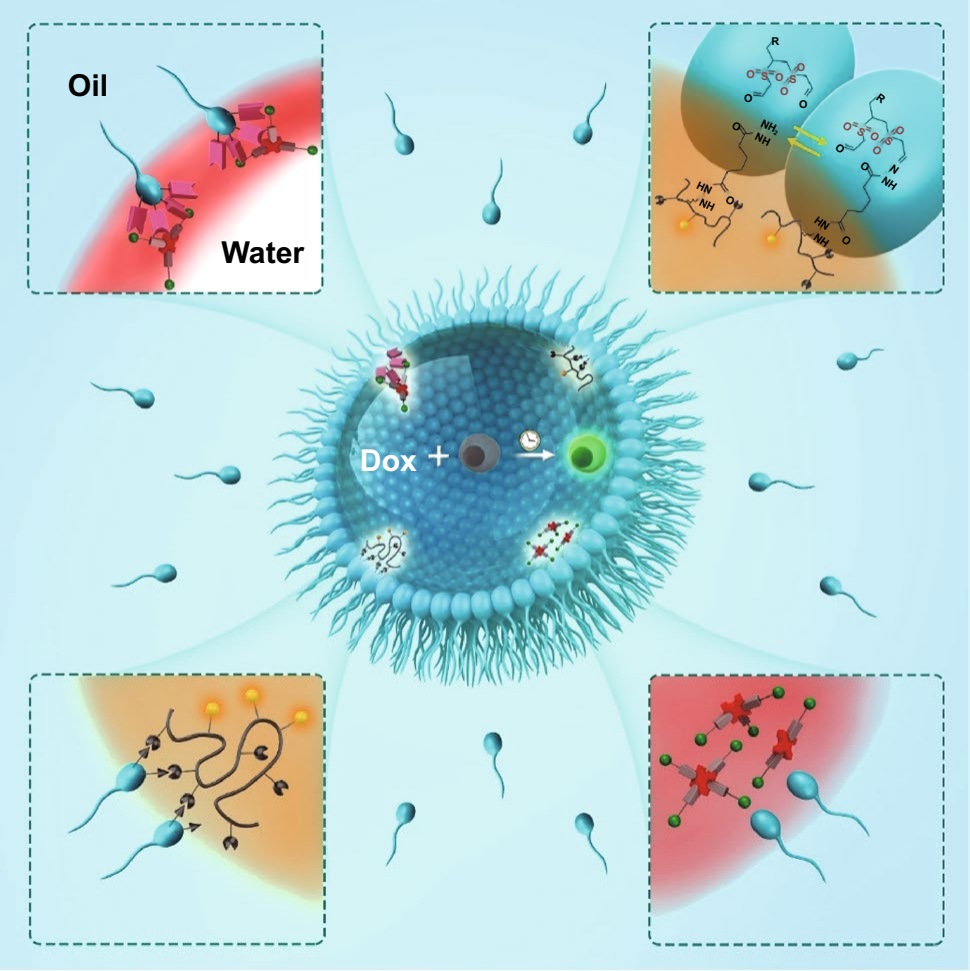
*Applications of functional surfactants* Scheme showing adsorption of surfactant molecules at the interface of oil (turquoise background) and water (surrounded by surfactants) to stabilize water-in-oil drop. The spherical part of the surfactant represents the polar head that meets water, while the wavy line represents the nonpolar tail that stays in the oil (turquoise background). At the center of the drop, it shows co-encapsulation of Doxorubicin (Dox) and a cell that yields a fluorescent cell. The top left inset shows the interaction of azide-bearing surfactant **4** with encapsulated proteins. The bottom left inset shows the interaction of aldehyde-bearing surfactant **5** with encapsulated polymer. The bottom right inset shows no interaction of non-functional surfactant **3** with the encapsulated proteins. An exemplary reversible chemical reaction is shown between aldehyde-bearing surfactant **5** and hydrazide-bearing polymer labeled with a fluorescent dye which is encapsulated into the drop (the top right inset, which corresponds to the bottom left inset)

Here, we combine a small, oxidation-responsive polar head group with a nonpolar perfluoropolyether (PFPE) tail to design a parent surfactant for creating a variety of multitasking surfactants that were easy to synthesize and scale-up (Fig. 1). By using droplet microfluidics, we further demonstrate how they can be leveraged for high-performance functional assays (Fig. 2). The surfactants exquisitely exploit different oxidation levels of thioethers, multivalency, tunability of -OH groups, and oxidizable 1,2-diols available in the polar part. We show the generality of the functional surfactants by using them for diverse droplet-based applications.

## Results and Discussion

### Synthetic Procedures

The synthesis of the polar surfactant part exquisitely benefits from the inexpensive, readily available propargyl amine and thioglycerol precursors, and rapid thiol-yne click chemistry. We performed the thiol-yne click reaction under UV light that allows two thiols to be integrated into one alkyne, creating a dendritic head with two thioethers and two 1,2-diols (Fig. S1). After acetal protection of the diols, we synthesized a parent surfactant through amide coupling between the activated PFPE tail and the amine-bearing head (Fig. S2). The successful coupling reaction was confirmed by the FT-IR signal at ~ 1720 cm^−1^ which corresponds to the amide peak (Fig. S3). The protection of the diols served three purposes: It prevented the side reaction between the -OH and the activated acid; it ensured selective oxidation of the thioethers; and it allowed better purification due to good solubility of the protected head groups in common organic solvents, whereas the conjugated pro-surfactant is exclusively soluble in fluorinated solvents. When the thioethers of the parent surfactant were selectively oxidized by either sodium periodate or meta-chloroperoxybenzoic acid they generated either sulfoxides or sulfones, respectively, in their backbones, leading to a dramatic increase in the polarity. Furthermore, the oxidation sensitivity of the novel head group was confirmed by ESI–MS (Figs. S4–S6), which would otherwise be difficult with the pro-surfactants or final surfactants due to solubility issues. Deprotection of the thioether-, sulfoxide-, and sulfone-containing surfactants under mildly acidic conditions created highly polar surfactants 1b, 2, and 3, respectively, all with four hydroxyl groups (Fig. 1). This posed opportunities for grafting a wide variety of polar and/or nonpolar functional molecules to the available -OH groups. As both sulfoxide and sulfone groups can highly enhance the overall polarity of the surfactants, the post-functionalization of the -OH groups with small nonpolar molecules should not affect the overall amphiphilic character of the surfactants.

### Functional Surfactants and Fishing of Biomolecules

**Fig. 3:**
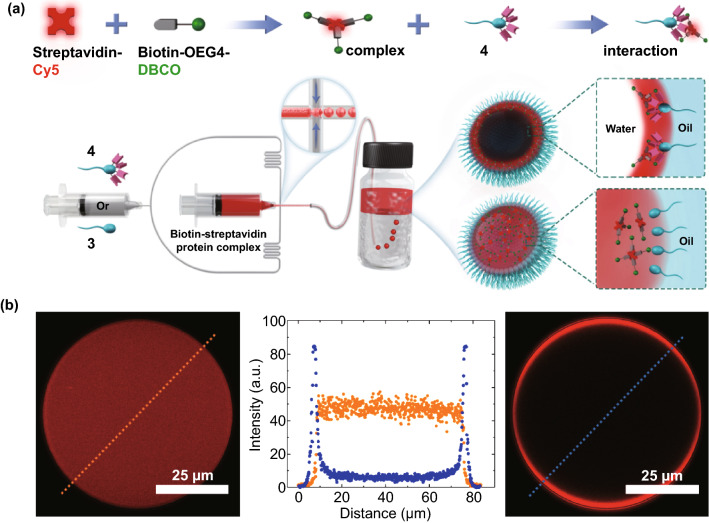
*Microfluidic approach to surfactant-stabilized drops for fishing biomolecules* **a** Mixing of Cy5 dye-labeled streptavidin (4-lock in red) with biotin (key in gray) connected via OEG4 (gray stick) to the strained cyclooctyne (green sphere) created a complex of biotin–streptavidin. This complex interacted with the surfactant 4, which contained azide groups (pink symbols) (the top panel). The bottom panel shows microfluidics generation of the complex-encapsulated drops using either surfactant 3 or 4 where the drops are pinched-off at the channel cross section (middle inset). The interior of the drop at the top inset shows the migration of complexes to the drop interface (successful fishing) with surfactant 4. By contrast, the interior of the drop at the bottom inset shows a homogeneous distribution of the complexes across the drop (no fishing) with surfactant 3. **b** 2D confocal fluorescent images of the complex-encapsulated drops and their fluorescence intensity profiles. The drops were stabilized by surfactant 3 (left) and surfactant 4 (right). In the intensity profiles plotted as a function of distance (middle), the orange line represents the spatial intensity of the drop of the left image and the blue one denotes the spatial intensity of the drop of the right image

To create a functional surfactant, we chose surfactant **3** as the starting surfactant as it had the maximum overall polarity, and it would allow grafting of azidoacetic acid to the -OH groups through facile esterification in one step (Fig. S2). The azide-functionalized surfactant **4** (Fig. 1) was characterized by FT-IR where two newly generated strong IR bands at ~ 1750 cm^−1^ and ~ 2100 cm^−1^ appeared, clearly indicating the corresponding ester and azide signals, respectively (Fig. S3). We then used droplet microfluidics to create water-in-oil emulsion droplets, which were stabilized either by surfactant **1b**, **2**, **3** or by the azido-surfactant **4** (Fig. S7). A 2% (w/w) solution of surfactant in HFE-7500 oil was injected into the microfluidic channel to create stable and monodisperse drops of ~ 70 µm in diameter (~ 200 pL) (Figs. S7–S8). The flow rates of the oil and aqueous phases were 1200 and 600 µL h^−1^, respectively. To conduct from-droplet fishing, the flow rates of the oil and aqueous phases were 600 and 300 µL h^−1^, respectively. The emulsion droplets were loaded with a protein complex comprised of Cy5 dye-labeled streptavidin and strained cyclooctyne-bearing DBCO-OEG4-Biotin which was pre-complexed in bulk before encapsulation into droplets. To prepare the protein complex, we employed 5 µM biotin and 0.63 µM streptavidin, providing a 2:1 ratio of biotin and biotin-binding site, respectively, and incubated them for ~ 3 h. Surprisingly, unlike PEG-based di-block surfactants [14], our di-block azido-surfactant could generate highly monodisperse droplets with an average diameter of 68.5 ± 0.9 µm (Fig. S9), justifying both the well-preserved amphiphilic character and the robustness of the functional surfactant, which can be unambiguously attributed to the highly polar sulfones in the backbone of the polar head. Furthermore, when the droplets were imaged after ~ 10 min, we found that the functional surfactants could effectively fish the protein complex from the droplet via rapid strain-promoted azide-alkyne cycloaddition (SPAAC) reaction between the surfactant and streptavidin–biotin complex. This resulted in the higher fluorescence intensity at the rim and no fluorescence signal within the drop (Figs. 3b and S9), clearly suggested that the azide-bearing surfactant enabled from-droplet fishing of the complexes. On the contrary, the surfactant **3** lacks azide functionality and could not fish the complex, resulting in a homogeneous distribution of the complex across the droplet (Figs. 3b and S9). It is noteworthy that biotin and streptavidin are widely used in biochemistry because of their robust and high affinity that dictates the design of numerous affinity-based assays [20, 21]. Moreover, many high-performance assays such as sandwich ELISA, single-cell barcoding, and single-cell antibody screening require sequential capture of target analytes where functional beads are used to immobilize them [6, 7, 22]. Having this in mind, we questioned whether our functional surfactant could pose any steric hindrance to the sequential fishing of biotin and streptavidin from drops and/or to fishing of the biotin–streptavidin complex that forms in drops. If not, skipping the complex formation in bulk should not be prohibitive to the from-droplet fishing of individual analytes or a complex comprised of multiple analytes. To validate this, we encapsulated biotin and streptavidin without pre-complexation into droplets that were stabilized with surfactant **4.** When the droplets were imaged at 1 h intervals for 3 h, we found that both droplet incubation and bulk incubation before encapsulation did not affect from-droplet fishing (Fig. S10), suggesting that our functional surfactant does not pose any limitations to protein activity and allows sequential fishing of the biotin and streptavidin from droplets. This feature indicates that our functional surfactant has a high potential to simplify the droplet-based assays requiring functional beads. It is worth mentioning that, before using DBCO-OEG4-Biotin, we used DBCO-Sulfo-Biotin to make the protein complex with the streptavidin-Cy5. Surprisingly, we found that from-droplet fishing was not successful (data not shown). The failure can be attributed to the linker type, which was also reported by others [23]. Hence, we believe our functional surfactant can also be a powerful tool in combination with droplet microfluidics for a fast, cheap, and effective screening of the activity of a wide variety of small-molecule linkers that are used in designing antibody–drug conjugate (ADC) [24] and digital single-biomolecule detection system [11].

### Capsule Fabrication

The robustness and intact surface activity of surfactant **4** motivated us to exploit the 1,2-diols in surfactant **3**, which is also responsive to oxidation. Since the highly polar sulfones in **4** contributed to the high degree of droplet stability and monodispersity, after oxidation of the diols in surfactant **3,** it should also enable the generation of highly monodisperse droplets with high stability due to sulfones. Furthermore, oxidation of the diols would create reactive aldehyde (-CHO) groups, which will bring another functionality for dynamic covalent chemistry. The dynamic covalent chemistry provides a powerful toolbox to generate reversible bonds with highly controllable reaction kinetics at different pH conditions via amine linkers with variable reactivities, such as alkoxyamine, carbazone, and hydrazide [25]. Conscious of these scopes, we oxidized the diols in **3** by sodium periodate to create surfactant **5**, bearing -CHO groups at the dendritic terminus which were characterized by FT-IR (Fig. S3). This allowed us to fabricate dynamic capsules in drops in one step using gelatin modified with adipic acid dihydrazide (ADH). It is worth mentioning that the creation of microcapsules in one step is a powerful way to protect the inner materials [8, 17, 26]. While the combination of droplet microfluidics and highly tunable kinetics of reversible chemistry can add advantages to generate highly monodisperse capsules with tunable degradation kinetics for pharmaceutics, perfume industries, and many other industrial applications.

**Fig. 4:**
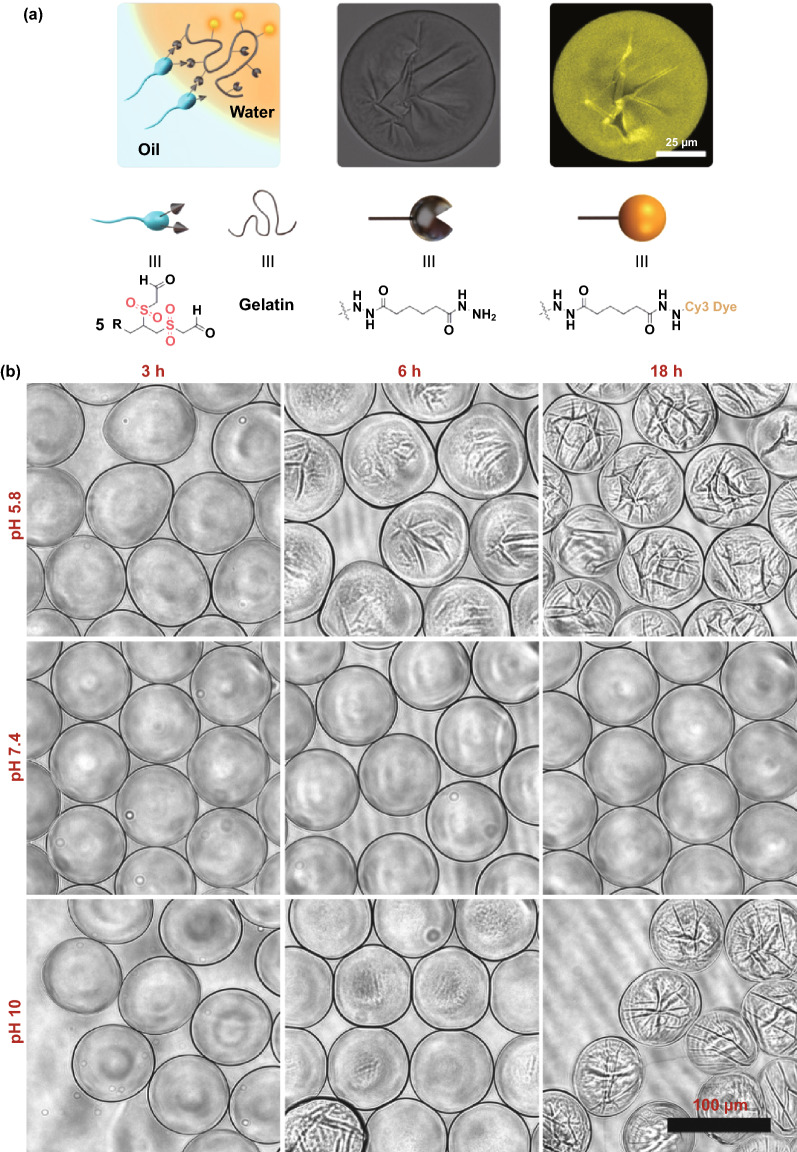
*One-step capsule fabrication* **a** Capsule formation scheme showing the reaction between the surfactant’s -CHO groups and the gelatin-ADH’s hydrazide groups at the water–oil interface of a drop (orange background). Drops were generated by using the single drop-maker PDMS device with two inlets for oil and aqueous solutions (used in Fig. 3A) and stabilized with surfactant **5** (left). 2D fluorescent (middle) and bright-field (right) images of a capsule were recorded with a confocal microscope after 24 h of droplet generation. The inner phase of the capsule comprised of 1% gelatin-ADH labeled with Cy-3 dye in the pH 10 borate buffer. The distribution of the fluorescent intensity across the capsule indicated that the polymer was not adsorbed at the interface due to the reversible hydrazone formation process across the capsule. **b** Time and pH-dependent capsule formation through reversible covalent bonds between 1% gelatin-ADH (in the aqueous phase) and the surfactant **5** (at the droplet interface)

We encapsulated 1% gelatin-ADH labeled with Cy-3 dye into droplets. We found that surfactant **5** was highly robust to generate monodisperse droplets and its aldehyde groups were well exposed to react with gelatin-ADH to form dynamic capsules (Fig. 4a). An analogous surfactant with ethers instead of sulfones could not create stable drops (Fig. S11), repeatedly validated the crucial role of sulfones in surfactants in maintaining the surface activity, stabilizing the droplets, and facilitating the cross-linking chemistry. To demonstrate the pH effect on hydrazone formation and therefore on the capsule creation, we encapsulated gelatin-ADH in three different pHs. We found that capsules started to form within 3 h and displayed varying morphology rather than being purely spherical at both acidic and basic conditions (Fig. 4b). While at physiological pH no change in their shape was detected, reflecting the usual slow hydrazone formation at neutral pH (Fig. 4b) [25]. However, long-term incubation till 18 h caused pronounced wrinkling at the capsule surface at both acidic and basic conditions unlike at neutral conditions, which we attributed to the increasing degree of hydrazone formation at the oil–water interface between surfactant’s aldehyde (-CHO) and gelatin-ADH’s hydrazide (Fig. 4b). Although we exploited only the reactivity of hydrazide that reacted with the aldehyde groups in our surfactants to form capsules, all other possible amine derivatives can also be exploited to fine-tune the reactivity on-demand and control the physicochemical properties of the capsules. Although we demonstrated the one-step capsule formation with the aldehyde-bearing head in the w/o system, the aldehyde-head can be used in any fluidic systems such as oil-in-water, water-in-oil-in-water, or more complex with variable hydrophobic tails.

### Single-cell Gene Expression in Droplets

Finally, to test the robustness and biocompatibility of the non-functional surfactants 1b, 2, and 3, we co-encapsulated the drug doxycycline (Dox) and Dox-inducible green fluorescent protein (GFP)-expressing Jurkat cells into droplets that were stabilized by these surfactants (Fig. S12). Testing of the surfactants 1b, 2, and 3 under the cell culture conditions revealed three crucial things: (i) the oxidized surfactants 2 and 3 generated more robust drops than the non-oxidized surfactant 1b even though all of them equally had four -OH groups, suggesting that oxidation of the thioethers was needed to increase polarity and enhance the robustness of the head group to secure the best performance of the surfactants; (ii) none of these surfactants interfered with the GFP expression at the single-cell level (Fig. S12), indicating that Dox could effectively activate the transcription system in the genome of the inducible cell to facilitate the gene expression; and (iii) cells could be easily released from droplets by washing surfactants with the fluorinated oil (Fig. S12).

## Conclusions

In summary, we have described five functional surfactants generated from one parent surfactant and their potential applications. The parent surfactant was synthesized using a nonpolar PFPE tail and an acetal protected novel small-molecule head obtained by an efficient thiol-yne-click reaction. The fluorinated tail enabled surfactants to be used for droplet microfluidics, while the head provided multivalency, readily adjustable functionality, and oxidation-responsive thioethers and 1,2-diols. These features of the small molecule were exploited to introduce multi-functionality to the surfactants and have a great potential to be exploited in designing anti-inflammatory nano-vehicles for biomedicine and in creating a broad range of high-performance polymers for diverse applications. The thioethers were oxidized to either sulfoxides or sulfones to add extra polarity to surfactants. The sulfone-bearing surfactant was used and by grafting azido-moieties to the -OH groups, we demonstrated the efficient fishing of biotin–streptavidin complexes from microfluidics-generated droplets via the efficient SPAAC reaction. This approach has the potential to replace functional beads that are used in droplet-based biochemical assays and to avoid plate-reader-based low-throughput and expensive assays for screening small-molecule linkers for ADC and beyond. By converting the 1,2-diols to -CHO groups, we depicted a one-step capsule fabrication at high-throughput using hydrazide-functionalized gelatin and aldehyde-bearing surfactant with sulfones at the backbone at different pH conditions. We showed that the physicochemical properties of the capsule could be easily controlled by varying the pH, which can be translated to other capsule systems by tuning the nonpolar tails and the reactivity of amine groups for creating capsules of various types for variable applications. Additionally, surfactants bearing -OH groups and either thioethers, sulfoxides, or sulfones exhibited excellent compatibility with the single-cell and its drug inducible gene expression in the drop and repeatedly justified the benefit of a higher degree of oxidation of thioethers by controlling microdroplet robustness. We envision that our approach to design functional surfactants will improve not only the basic research and development but also a broad range of applied sciences.

## Supplementary Information

Below is the link to the electronic supplementary material.

Supplementary file1 (DOCX 787 kb)

## References

[1] Urner LH, Liko I, Yen H-S, Hoi K-K, Bolla JR (2020). Modular detergents tailor the purification and structural analysis of membrane proteins including G-protein coupled receptors. Nat. Commun..

[2] Tang Z, Kong N, Zhang X, Liu Y, Hu P (2020). A materials-science perspective on tackling COVID-19. Nat. Rev. Mater..

[3] Theberge AB, Courtois F, Schaerli Y, Fischlechner M, Abell C (2010). Microdroplets in microfluidics: an evolving platform for discoveries in chemistry and biology. Angew. Chem. Int. Ed..

[4] Liu L, Xiang N, Ni Z, Huang X, Zheng J (2020). Step emulsification: high-throughput production of monodisperse droplets. Biotechniques.

[5] Agresti JJ, Antipov E, Abate AR, Ahn K, Rowat AC (2010). Ultrahigh-throughput screening in drop-based microfluidics for directed evolution. Proc. Natl. Acad. Sci. USA.

[6] Klein AM, Mazutis L, Akartuna I, Tallapragada N, Veres A (2015). Droplet barcoding for single-cell transcriptomics applied to embryonic stem cells. Cell.

[7] Gérard A, Woolfe A, Mottet G, Reichen M, Castrillon C (2020). High-throughput single-cell activity-based screening and sequencing of antibodies using droplet microfluidics. Nat. Biotechnol..

[8] Zhang J, Coulston RJ, Jones ST, Geng J, Scherman OA (2012). One-step fabrication of supramolecular microcapsules from microfluidic droplets. Science.

[9] Miller TE, Beneyton T, Schwander T, Diehl C, Girault M (2020). Light-powered CO_2_ fixation in a chloroplast mimic with natural and synthetic parts. Science.

[10] Weiss M, Frohnmayer JP, Benk LT, Haller B, Janiesch J-W (2018). Sequential bottom-up assembly of mechanically stabilized synthetic cells by microfluidics. Nat. Mater..

[11] Cohen L, Cui N, Cai Y, Garden PM, Li X (2020). Single molecule protein detection with attomolar sensitivity using droplet digital enzyme-linked immunosorbent assay. ACS Nano.

[12] Holtze C, Rowat AC, Agresti JJ, Hutchison JB, Angilè FE (2008). Biocompatible surfactants for water-in-fluorocarbon emulsions. Lab Chip.

[13] Chowdhury MS, Zheng W, Kumari S, Heyman J, Zhang X (2019). Dendronized fluorosurfactant for highly stable water-in-fluorinated oil emulsions with minimal inter-droplet transfer of small molecules. Nat. Commun..

[14] Platzman I, Janiesch J-W, Spatz JP (2013). Synthesis of nanostructured and biofunctionalized water-in-oil droplets as tools for homing T cells. J. Am. Chem. Soc..

[15] Cui M, Emrick T, Russell TP (2013). Stabilizing liquid drops in nonequilibrium shapes by the interfacial jamming of nanoparticles. Science.

[16] Huang C, Forth J, Wang W, Hong K, Smith GS (2017). Bicontinuous structured liquids with sub-micrometre domains using nanoparticle surfactants. Nat. Nanotechnol..

[17] Kumar D, Paulsen JD, Russell TP, Menon N (2018). Wrapping with a splash: high-speed encapsulation with ultrathin sheets. Science.

[18] Zarzar LD, Sresht V, Sletten EM, Kalow JA, Blankschtein D (2015). Dynamically reconfigurable complex emulsions via tunable interfacial tensions. Nature.

[19] Yang Z, Wei J, Sobolev YI, Grzybowski BA (2018). Systems of mechanized and reactive droplets powered by multi-responsive surfactants. Nature.

[20] Cheung AS, Zhang DKY, Koshy ST, Mooney DJ (2018). Scaffolds that mimic antigen-presenting cells enable ex vivo expansion of primary T cells. Nat. Biotechnol..

[21] Rissin DM, Kan CW, Campbell TG, Howes SC, Fournier DR (2010). Single-molecule enzyme-linked immunosorbent assay detects serum proteins at subfemtomolar concentrations. Nat. Biotechnol..

[22] Wu C, Garden PM, Walt DR (2020). Ultrasensitive detection of attomolar protein concentrations by dropcast single molecule assays. J. Am. Chem. Soc..

[23] Cohen L, Walt DR (2018). Evaluation of antibody biotinylation approaches for enhanced sensitivity of single molecule array (Simoa) immunoassays. Bioconj. Chem..

[24] Lyon RP, Bovee TD, Doronina SO, Burke PJ, Hunter JH (2015). Reducing hydrophobicity of homogeneous antibody-drug conjugates improves pharmacokinetics and therapeutic index. Nat. Biotechnol..

[25] Boehnke N, Cam C, Bat E, Segura T, Maynard HD (2015). Imine hydrogels with tunable degradability for tissue engineering. Biomacromolecules.

[26] Amstad E (2018). Capsules made from prefabricated thin films. Science.

