# Supplementary material for: Functional Surfactants for Molecular Fishing, Capsule Creation, and Single-Cell Gene Expression
Source: Nanomicro Lett. 2021 Jun 19;13:147. doi: 10.1007/s40820-021-00663-x (PMC8214653; doi:10.1007/s40820-021-00663-x)
Supplement: Supplementary file 1 — Supplementary file1 (DOCX 787 kb) [file 40820_2021_663_MOESM1_ESM.docx]

Supporting Information for

Functional Surfactants for Molecular Fishing, Capsule Creation, and Single-Cell Gene Expression

Mohammad Suman Chowdhury^1,^ *^,^ ^†^, Xingcai Zhang^2,^ ^3,^ ^†^, Leila Amini^4,5^, Pradip Dey^1^, Abhishek Kumar Singh^1^, Abbas Faghani^1^, Michael Schmueck-Henneresse^4,5^, Rainer Haag^1,^ *

^1^Institut für Chemie und Biochemie, Freie Universität Berlin, Takustrasse 3, 14195 Berlin, Germany

^2^John A. Paulson School of Engineering and Applied Sciences, Harvard University, Cambridge, Massachusetts 02138, USA

^3^School of Engineering, Massachusetts Institute of Technology, Cambridge, Massachusetts 02139, USA

^4^Berlin Institute of Health – Center for Regenerative Therapies, Charité Universitätsmedizin Berlin – CVK, Föhrer Str. 15, 13353 Berlin

^5^Berlin Center for Advanced Therapies, Charité Universitätsmedizin Berlin – CVK, Föhrer Str. 15, 13353 Berlin

†Mohammad Suman Chowdhury and Xingcai Zhang are co-first authors

*Corresponding authors. E-mail: [te.suman07012@gmail.com](mailto:te.suman07012@gmail.com) (Mohammad Suman Chowdhury), [haag@chemie.fu-berlin.de](mailto:haag@chemie.fu-berlin.de) (Rainer Haag)

# S1 Materials and Methods

## S1.1 Materials

Monocarboxylic acid-bearing Krytox 157-FSL (MW = ~2200 g mol-1) was purchased from LUB SERVICE GmbH (Germany). Both HFE 7100- and HFE 7500-fluorinated oils were bought from 3M. Dibenzylcyclooctyne (DBCO)-OEG4-Biotin and DBCO- Sulfo-Biotin were obtained from Jena Bioscience (Germany). All other chemicals we purchased were reagent grade. These chemicals were from Acros Organics (Belgium) and/or from Merck (Germany) unless otherwise mentioned. They were used as received without further purification. For moisture-sensitive synthesis, flame-dried glasswares were used. All reactions were performed under an inert atmosphere. For droplet generation, we prepared 2% (w/w) surfactant solutions in HFE-7500 oil irrespective of surfactant types.

## S1.2 Instrumentation

We used an AMX 500 spectrometer (Bruker, Switzerland) to obtain the NMR spectra. δ values in ppm were used to report the chemical shift of the proton NMR. For peak calibration, we used deuterated solvent peaks. For UV-irradiation, we used a 100 W Ushio (Tokyo, Japan) USH-102d mercury short arc lamp. The FT-IR spectra were recorded from 4000 to 650 cm^-1^ wavenumbers by Nicolet AVATAR 320 FT-IR 5 SXC (Thermo Fisher Scientific, USA) with a DTGS detector. We took fluorescence images using the Zeiss microscope (Zeiss, Germany) and the Leica confocal microscope (TCS SP8, Germany). During water-in-oil (w/o) emulsion droplet generation by microfluidic flow focusing, we used a high-resolution CCD camera with 3 MPixel (Jena, Germany) for brightfield imaging. We used OriginPro 2019b (academic version) to plot all the FT-IR spectra. We used Image J to prepare a fluorescence intensity profile from the spatial intensity of fluorescent droplets.

## S1.3 Synthesis of Compound

### S1.3.1 Synthesis of compound 1aꞌ

To a dry 250 mL round-bottom flask, propargyl amine (5 g, 90.8 mmol), thioglycerol (29.5 g, 272.3 mmol), and 2,2-dimethoxy-2-phenylacetophenone (DMPA) photoinitiator (0.93 g, 3.63 mmol) were added. The thiol-yne click chemistry was performed following a literature procedure with some modifications [S1]. In short, the chemicals were thoroughly mixed rotating using a rotavapor while heating at 40 °C without applying vacuum under dark conditions. Before irradiating with the 365 nm UV-light, the flask was flushed with argon and kept under inert conditions using an argon-filled balloon. The contents were then irradiated at room temperature (RT) with 365 nm UV-light at 60 W without stirring. Subsequently, the crude product was dissolved in a minimum amount of methanol and precipitated into excess diethyl ether (2x). The crude was then dried under reduced pressure and without further purification and used for the protection of 1,2-diols by the acetal protecting group.

The acetal protection was conducted following the reported method with a slight modification [S2]. 2,2- dimethoxypropane (0.73 mol, 75.63 g) and p-toluene sulfonic acid monohydrate (pTSA.H2O) (9.99 mmol, 18.99 g) were added to the crude product. The mixture was then stirred overnight at RT. The reaction was quenched by adding triethylamine (13.77 g, 136 mmol). Then the solvent was evaporated in rotavapor.

Next, the residue was suspended in water and dichloromethane (3×100 mL). The organic layers were combined and dried over sodium sulfate and then the solvent was evaporated to yield the crude product, which had been purified using column chromatography with DCM and methanol to give compound **1aꞌ** (**Fig. S1**) as a brown viscous liquid with 30% isolated yield (~9.6 g). ^1^H NMR (500 MHz, CDCl3) for compound **1aꞌ**: δ 4.32- 4.21 (p, 2H), 4.15-4.04 (dd, 2H), 3.82-3.56 (dd, m, 4H), 3.12-

2.62 (m, 7H), 1.45-1.40 (s, 6H), 1.38-1.31 (s, 6H); ESI-MS calculated m/z for C15H30NO4S2 [M+H]^+^: 352.1538, found: 352.1584) (**Fig. S4**).

### S1.3.2 The general procedure to prepare sulfoxide derivative (compound 2aꞌ)

Oxidation of thioether to sulfoxide was performed following the reported procedures with some modifications [S3]. To a vial equipped with a magnetic stirrer bead, compound **1aꞌ** (100 mg, 0.28 mmol) and NaIO_4_ (1.2 g, 5.68 mmol) were added. Then 2 mL of THF and water (1:1) mixture was added to it. The reaction mixture was vigorously stirred overnight at RT. Selective oxidation of the thioethers to the corresponding sulfoxides (**Fig. S1**) was monitored by ESI- MS (**Fig. S5**). ESI-MS calculated m/z for C15H30NO6S2 [M+H]^+^: 384.1436, found: 384.1479).

### S1.3.3 The general procedure to prepare sulfone derivative (compound 3aꞌ)

Oxidation of thioether to sulfone was performed following the reported procedures with some modifications [S4]. To a vial equipped with a magnetic stirrer bead, compound **1aꞌ** (100 mg, 0.28 mmol) and mCPBA (320 mg, 1.82 mmol) were added. Then ~6 mL dimethylformamide (DMF) was added to it. The reaction mixture was vigorously stirred overnight at RT. Selective oxidation of the thioethers to the corresponding sulfones (**Fig. S1**) was monitored by ESI- MS (**Fig. S6**). ESI-MS calculated m/z for C15H30NO8S2 [M+H]+: 416.1335, found: 416.1290).

## S1.4 Synthesis of Surfactant

### S1.4.1 Synthesis of parent surfactant (PS) 1a

The di-block **‘PS’ 1a** was synthesized in a two-step process (**Fig. S2**) following the

reported protocols with some modifications [S2, S5]. In the first step, an acid chloride derivative of PFPE- COOH was prepared by dropwise addition of thionyl chloride (1.62 g, 13.63 mmol) under ice- cooled condition in a mixture of diisopropylethylamine (DIPEA) (1.37 g, 13.63 mmol) and PFPE-COOH (15 g, ~6.82 mmol) in HFE-7100 (30 mL) taken in a 100 mL round-bottomed flask equipped with magnetic stirrer bead. After thionyl chloride addition, the ice-bath was removed, and the reaction continued overnight at RT with continuous stirring under an inert atmosphere. To remove the generated salt, the crude mixture was filtered through cotton wool and dried under reduced pressure. The residue was then redissolved in 30 mL of HFE-7100. In the second step, compound **1aꞌ** was reacted with the activated PFPE. Compound **1aꞌ** (4.79 g, 13.63 mmol) and DIPEA (1.37 g, 13.63 mmol) were dissolved in DCM and then added dropwise into the solution of activated-PFPE. In summary, 30 mL DCM was added to provide better miscibility with HFE-7100 oil.

The reaction mixture was then refluxed at 50 °C overnight. The crude reaction mixture was dried under reduced pressure and washed with methanol (5x30 mL). Thus, all the unreacted head groups (**1aꞌ**) and possible by products were removed during washing, providing the di-block **‘PS’ 1a** with the protected hydroxyl groups. The isolated yield of the **‘PS’ 1a** was ~85%.

### S1.4.2 Synthesis of surfactant 1b

Deprotection of the acetal groups in the **‘PS’ 1a** was performed overnight under reflux condition using 1.25% (w/v) HCl in methanol-HFE7100 solvent mixture, which created the water-soluble polar head group in the surfactant. The crude fluorosurfactant **1b** was then washed with methanol (3×30 mL, for a 5 g batch) to remove the acid and obtain a ~80% isolated yield after drying under reduced pressure.

### S1.4.3 Synthesis of surfactants 2 and 3

Surfactants **2** and **3** were synthesized in two steps. Firstly, the thioethers in the **‘PS’ 1a** were selectively oxidized to either sulfoxides or sulfones following the *general oxidation procedures* described above with slight modification in solvent composition to ensure better miscibility of the reactants. For oxidation of thioethers to sulfoxides, a solvent mixture comprised of H_2_O, CH_3_OH, and HFE7100 was used. For a 5 g batch, a total of ~20 mL solvent was used where these solvents were mixed at a ratio of 4:6:10, respectively. For oxidation of thioethers to sulfones, a mixture of DMF and HFE7100 (1:1) was used. For a 5 g batch, ~20 mL solvent was used. Next, the oxidized pro-surfactants were treated with mild acidic conditions for the deprotection of the acetal groups. Refluxing them at 50 °C overnight in the 1.25% (w/v) HCl in

methanol-HFE7100 solvent mixture led to the generation of either surfactant **2** or surfactant **3** containing the water-soluble polar head groups in surfactants. The crude fluorosurfactants were then washed with methanol (3x30 mL, for a 5 g batch) to remove the acid and both obtained with ~85% isolated yields after drying them under reduced pressure.

### S1.4.4 Synthesis of surfactant 4

Surfactant **4** was prepared in one step. To a 50 mL round bottom flask equipped with a magnetic stirrer bead, surfactant **3** (300 mg, 0.14 mmol), azido acetic acid (68.9

mg, 0.68 mmol), EDC. HCl (195.03 mg, 1.02 mmol), and DMAP (73.3 mg, 0.6 mmol) were added. Then 10 mL solvent consisting of a mixture of DMF and HFE7500 oil (1:1) were added to it. The mixture was vigorously stirred overnight at 45 °C . When stirring was stopped, within minutes, DMF was phase separated. After removing the

DMF part, the fluorous oil phase was washed with pure DMF (3×10 mL), which removed the reactants and by-products soluble in DMF. After drying under reduced pressure, azido fluorosurfactant **4** was obtained with a ~89% isolated yield (~316 mg).

### S1.4.5 Synthesis of surfactant 5

Surfactant **5** was prepared in one step. To a 100 mL round bottom flask equipped with a magnetic stirrer bead, surfactant **3** (1.2 g, 0.91 mmol) and NaIO4 (3.9 g, 18.18 mmol) were added. Then 10 mL solvent consisting of a mixture of water, methanol, and HFE7100 oil at a 2:3:5 ratio, respectively, was added to it. The reaction mixture was vigorously stirred overnight at RT. The aqueous phase was carefully removed by washing with methanol (3×10 mL). Then ~10 mL HFE7500 was added, which facilitated the precipitation of residual NaIO4 and other impurities generated during the reaction. The supernatant was collected and filtered using a double-layer cellulose filter paper. After drying under reduced pressure, fluorosurfactant **5** with aldehyde functionality was obtained with a ~40% isolated yield (~0.48 g).

## S1.5 PDMS Device Fabrication

The single-drop-making PDMS device was prepared according to our reported protocol with no further modification [S2].

## S1.6 Preparation of Biotin and Streptavidin Solutions for Droplet Encapsulation

For the from-droplet-fishing assay, stock solutions of DBCO-OEG4-Biotin and Cy®5- streptavidin were prepared following the suppliers’ guidelines. Milli-Q (MQ) water was used to prepare a working solution of each compound. For droplet incubation, DBCO-OEG4-Biotin and Cy®5-streptavidin were mixed at a molar ratio of 8:1 to make up a final concentration of 5 µM:0.63 µM, respectively, in 500 µL MQ water. Of note, this ratio provided two biotin molecules for each biotin-binding site in the streptavidin which had four biotin-binding sites in total. The solution was then vortexed for 30 s and then immediately transferred into a 1 mL syringe for droplet encapsulation. On the contrary, for bulk incubation prior to droplet encapsulation, three samples were prepared separately containing same amounts of biotin and streptavidin in 500 µL MQ water. These freshly prepared solutions were stirred in 2

mL glass vials, equipped with magnetic stirrer beads, at 200 rpm at RT for 1, 2, and 3 h, respectively. Each sample was then transferred into a 1 mL syringe at the indicated time point for droplet encapsulation. The flow-focusing nozzle used in the single droplet maker was 35 µm × 50 µm. The flow rates of 600 µL/h for the oil phase and 300 µL/h for the aqueous phase generated ~80 µm droplets. For droplet generation, a 2% surfactant (w/w) solution in HFE 7500 oil was used.

## S1.7 Gelatin Functionalization and Capsule Fabrication

The commercially available gelatin (type A) was functionalized with adipic acid dihydrazide (ADH) following a literature procedure to prepare gelatin-ADH where all the carboxylic acid groups were modified with the ADH [S6]. To prepare Cy3 dye- labeled gelatin-ADH, 3.5 mg (~4.5 µM) of N HS-activated Cy3 dye was added to a solution of 250 mg gelatin-ADH (~460 µM of ADH) dissolved in ~2.5 mL of deionized (DI) water and diisopropylamine (DIPEA). Additionally, ~2.5 mL DMSO was added to ensure better miscibility of the dye. This reaction was performed overnight at 45 °C followed by dialysis in water for 2 days at 40 °C . A regenerated cellulose (RC) dialysis membrane with a molecular weight cut-off of 3500 Da was used for dialysis. After drying the solution under reduced pressure, ~200 mg dye-

labeled gelatin-ADH was obtained which was stored at 4 °C for further use.

To fabricate capsules at different pH conditions, a 1% gelatin-ADH solution was prepared in DI water at varying pHs. A 2% (w/w) solution of the -CHO bearing surfactant **5** in HFE-7500 was employed to generate droplets loaded with the gelatin solution. A single droplet maker device with two inlets (one for oil and one for aqueous media) was used as demonstrated in the main text (**Fig. 3a**). The flow rates of the aqueous and oil phases were 300 and 600 µL/h, respectively. The generated droplets were incubated for varying time points at ~25 °C and imaged using a confocal microscope to monitor the progress of the capsule formation.

## S1.8 Stable DOX-inducible Jurkat Cell Line Generation

Jurkat cells were cultured in very low endotoxin-containing Roswell Park Memorial Institute (RPMI) 1640 medium under standard cell culture conditions. The medium was supplemented with 10% (v/v) fetal bovine serum (FBS) and 1% (v/v) penicillin- streptomycin (P-S). To generate a stable Tet-On 3G doxycycline (Dox)-inducible green fluorescence protein (GFP)- expressing reporter-cell line, Jurkat cells were transfected with hyperactive *piggyBac* transposase (pCMV-hyPBase, a kind gift from the Sanger Institute, UK) [S7] and XLone-GFP (a gift from Xiaojun Lian; Addgene plasmid # 96930) [S8] plasmid constructs. Program CL-120 of the 4D Nucleofector (Lonza) was used to electroporate the Jurkat cells with 10 µg of the XLone-GFP and 25 µg of the pCMV-hyPBase plasmids. The plasmids and 1 million cells were dissolved in 100 µL electroporation buffer P3 before electroporation. This solution was transferred into a 48 wells plate prefilled with a 1 mL prewarmed medium. On the following day, the cells were transferred into T25 flasks and then 4 mL of fresh medium was added. On day 5, cells were treated with 4 µM Dox to turn on the green fluorescent protein (GFP) expression. On day 8, FACS was used to isolate a pure population of cells having the maximum GFP fluorescence intensity. The FACS sorted cells were then grown for three days and frozen in a freezing medium (FBS + 10% DMSO).

# S2 Supplementary Figures

**
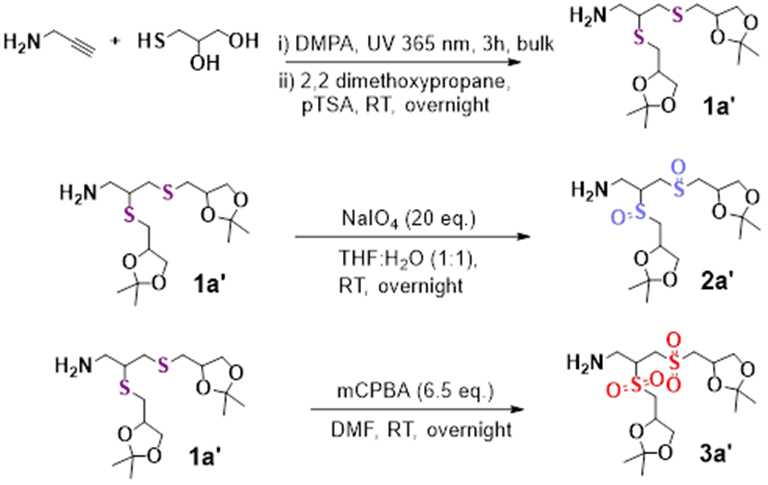
**

**Fig. S1** Schematic synthetic approach to compounds 1aꞌ-3aꞌ through thiol-yne click reaction, acetal protection, and oxidations


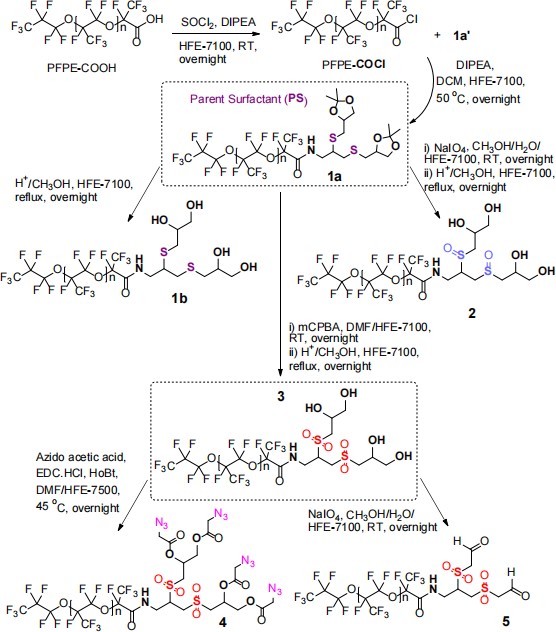


**Fig. S2** Schematic synthetic approach to surfactants 1b, 2, 3, 4, and 5 through the synthesis of the parent surfactant (PS) 1a and subsequent oxidations and post- functionalization


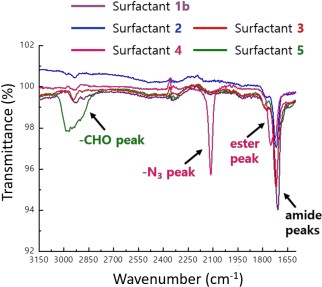


**Fig. S3** Characterization of the surfactants by FT-IR

FT-IR spectra showing the presence of amide signals in the surfactants 1b, 2, 3, 4 and 5 at ~1720 cm^-1^. The successful preparation of the azido acetic acid coupled surfactant 4 was confirmed by both the ester signal at ~1750 cm^-1^ and the azide signal at ~2100 cm^-1^. Surfactant 5, which was created by oxidizing the 1,2-diols, showed a new signal at ~2850 cm^-1^, which confirmed the formation of -CHO groups after the oxidation


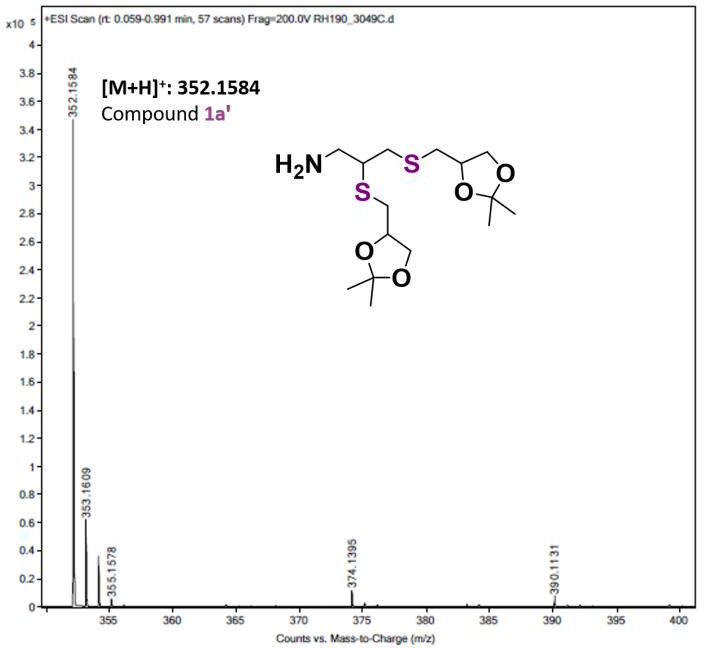


**Fig. S4** ESI-MS of compound **1aꞌ**


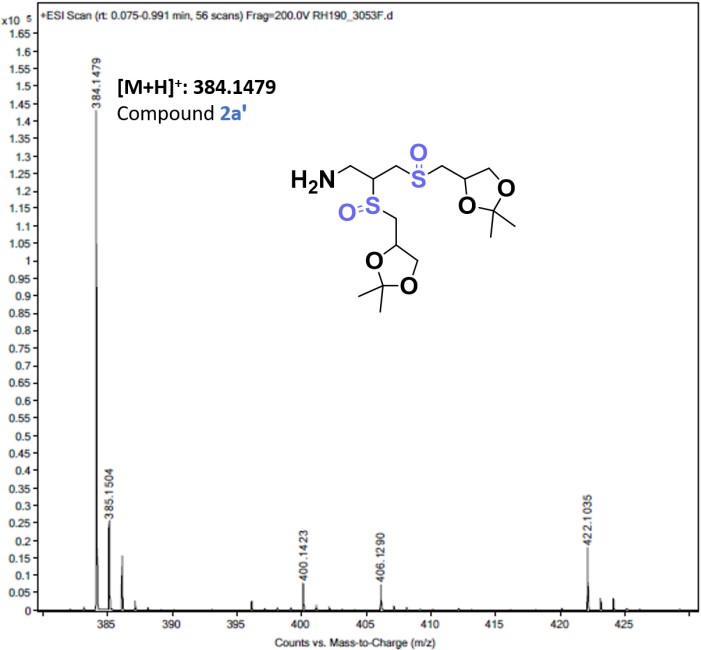


**Fig. S5** ESI-MS of compound **2aꞌ**


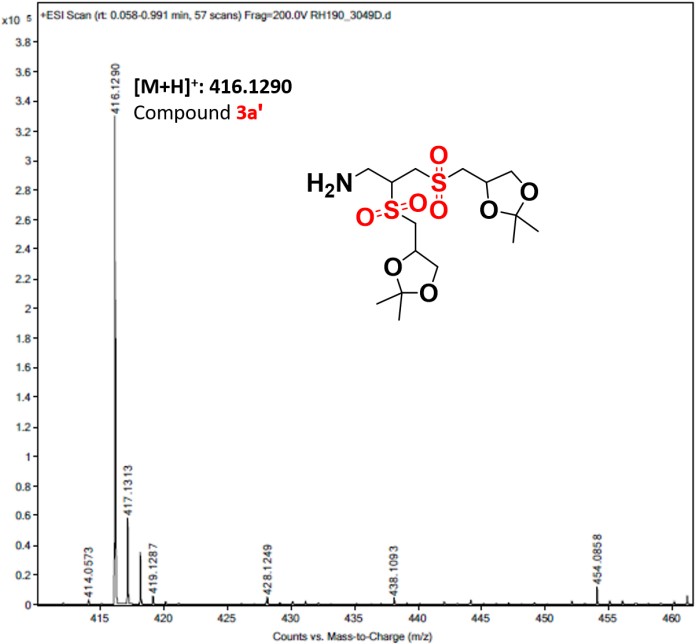


**Fig. S6** ESI-MS of compound **3aꞌ**


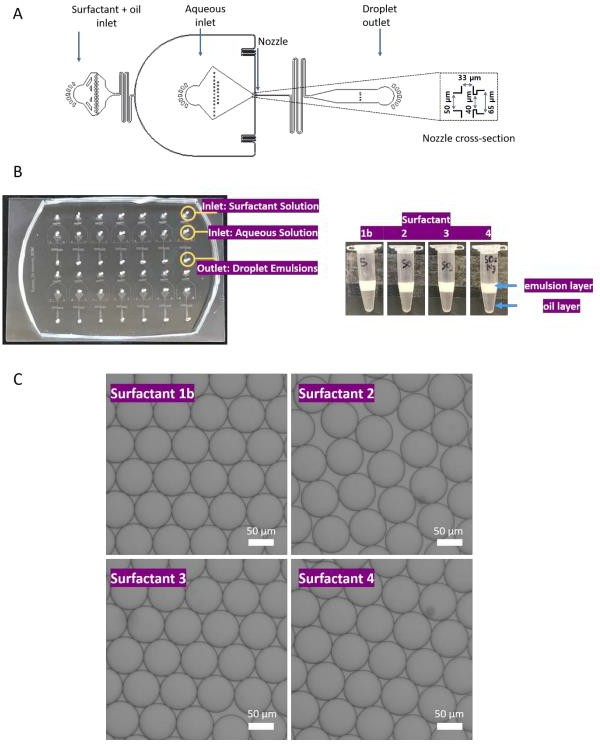


**Fig. S7** (**A**) A microfluidic approach for single droplet generation. Dimension of the nozzle cross-section is shown on the inset (right). The height of the channel was 50 µm. The 15 mm long serpentine after the nozzle was used to allow the surfactant molecules to better adsorb at the water-oil interface. (**B**) Image showing a single droplet making PDMS device (left). The PDMS device has 14 single droplet making channels each with one inlet for aqueous stream, one inlet for surfactant solution, and one outlet for droplet collections. The inlet and outlet of the channel were connected to syringe needles via PE20 tubing (dimension: I.D. 0.38 mm, O.D. 1.09 mm; order no. 427406; BECTON DICKINSON). Emulsions were collected in Eppendorf tubes where the white layer contains the emulsion droplets, and the residual surfactant solution (transparent) remains underneath the emulsion layer because of the high density of HFE7500 oil (ρ=1.614 g/mL) (right). (**C**) Micrographs showing the representative droplets stabilized by surfactants 1b, 2, 3, and 4. The oil and aqueous flow rates were 1200 µL/h and 600 µL/h respectively while generating the droplets.


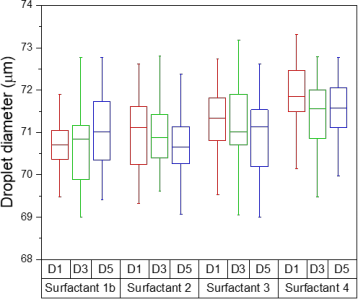


**Fig. S8** Box-plot of droplet size distribution

The droplets were incubated at room temperature and imaged at days 1 (D1), 3 (D3), and 5 (D5) for the size distribution analysis. The aqueous phase contained PBS buffer. A total of 50 droplets were analyzed by ImageJ for each surfactant. The box plots represent the median (center line), mean ± s.d. (box), and mean ± 1.5 x s.d. (whisker).


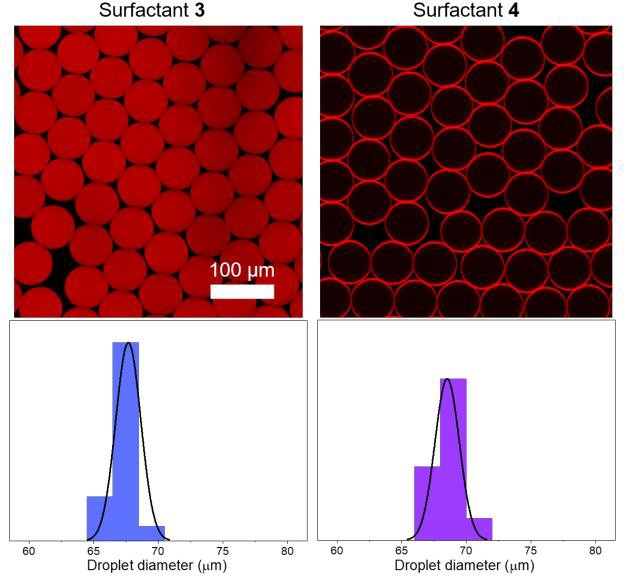


**Fig. S9** From-droplet biomolecules’ fishing and the droplet-size distribution

Confocal fluorescent images of the droplets loaded with biotin-streptavidin complexes (top panel). These complexes were prepared in bulk for ~3 h by mixing DBCO- OEG4-Biotin (8 eq.) with Cy®5- streptavidin (1 eq.). The final concentrations of the biotin and streptavidin molecules were 5 µM and 0.63 µM, respectively. Droplets were stabilized with surfactant 4 (top left) and surfactant 3 (top right). Unlike surfactant 3, surfactant 4 enabled from-droplet fishing of the protein complexes, as indicated by the strong fluorescence intensity at the rim of the droplets and no fluorescence intensity within the droplets (top left). The droplets were imaged after 10 minutes of droplet generation. The size distribution of the droplets (bottom panel) showed that highly monodisperse droplets were generated by both surfactant 3 (bottom right) and surfactant 4 (bottom left), which suggested that both the modified 4

and non-modified 3 surfactants were equally good to create highly stable and highly monodisperse droplets for the assay. In addition, it suggested that the sulfones in the backbone of the head group allowed the surfactant 4 to maintain its surface activity even though the hydroxyl groups were functionalized with the non-polar azido moieties.


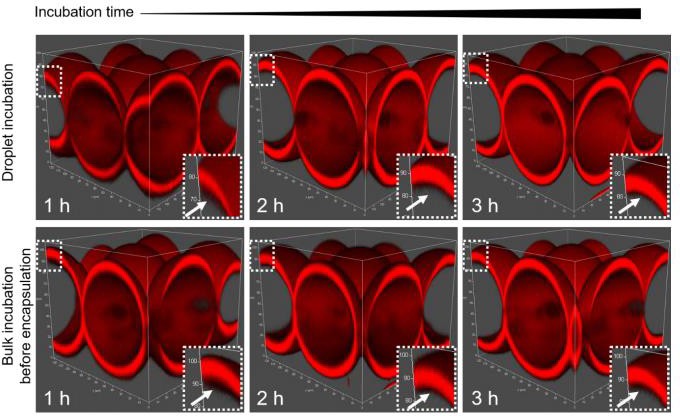


**Fig. S10** Influence of droplet vs. bulk incubation of proteins on fishing efficiency. Here, bulk incubation refers to making the biotin-streptavidin complex outside the drops, while droplet incubation refers to making the complex inside drops, which poses an opportunity for both sequential fishing of individual proteins and fishing of the complex. The insets show the from-droplet fishing kinetics by means of reduced thicknesses of black areas (denoted by white arrows) under both incubation conditions at the indicated time points. Droplet incubation enabled slightly better fishing than bulk incubation at 2 h and 3 h, revealing that sequential fishing and fishing the complex only both can be done without compromising with the fishing efficiency. In those images, droplets were stabilized with surfactant 4 and the aqueous phase comprised 5 µM DBCO-OEG4-Biotin and 0.63 µM streptavidin-Cy5.


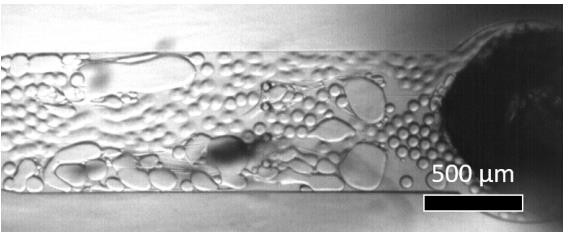


**Fig. S11** Performance of 1,2-diols oxidized surfactant lacking thioethers

We used our previously reported surfactant L-dTG [S2], which had two 1,2-diols as in surfactant 1 but lacking thioethers in the backbone. We chose this to prepare an analogous surfactant like surfactant 5 but without the possibility to have sulfones in the backbone. This allowed us to justify the role of sulfones in creating and stabilizing the droplets with surfactant-lacking hydroxyl groups and to compare its performance with surfactant 5. Oxidation of the 1,2-diols with sodium metaperiodate was conducted following the procedures discussed above. We found that the 1,2-diols oxidized surfactant L-dTG failed to generate stable droplets even though it had a similar length and architecture as in surfactant 5. The image of the unstable droplets was taken during droplet generation. Note, some droplets were seen to be monodisperse as shown in the figure, but they coalesced shortly after collection.


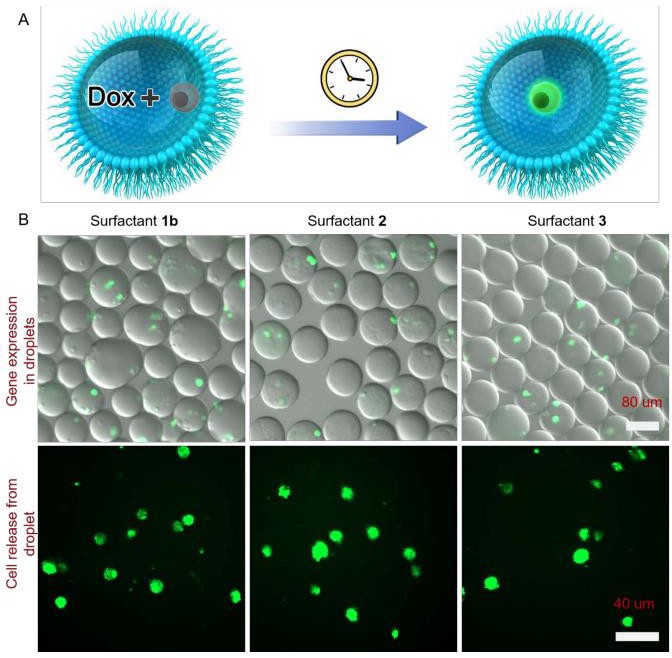


**Fig. S12** Drug-inducible gene expression in single-cell cultured in droplets. (**A**) Schematic showing the co-encapsulation of the drug Dox and a non-fluorescent single-cell (grey) (within the aqueous phase) after droplet generation. The droplet was stabilized with either surfactant 1b, surfactant 2, or surfactant 3 as indicated in the images. The cell (green) started to express the GFP gene with time as Dox activated its transcription system to facilitate the gene expression. (B) Prior to cell encapsulation into droplets, Dox-inducible cells were dispersed in RPMI 1640 medium containing 4 µg/mL Dox, 5 µg/mL BSD, and 17% (v/v) Opti-prep. The cell density was 6.0 x 10^6^ cells/mL, and the cell culture medium was supplemented with 10% FBS and 1% P-S. A single-droplet-maker device with two inlets (one for oil and one for cell culture media with cell suspension) was used as demonstrated in the main text (Fig. 3A). The flow rates for droplet generation were 600 µL/h and 300 µL/h for

oil and aqueous phases, respectively. The droplets were incubated for 48 h to turn on the GFP expression and, subsequently, differential interference contrast (DIC) images of the droplets were taken using a fluorescence microscope. To release the encapsulated cells from 100 µL emulsion droplets, the droplets were washed with HFE-7500 oil (10x500 µL) until the droplets coalesced into a single aqueous layer.

After each wash, the excess oil underneath the emulsion and/or aqueous layer was removed by a syringe mounted with a long needle. The released cells were then imaged using a fluorescence microscope. Of note, the contaminated HFE-7500 oil can be easily recycled by distillation using a rotavapor under reduced pressure.


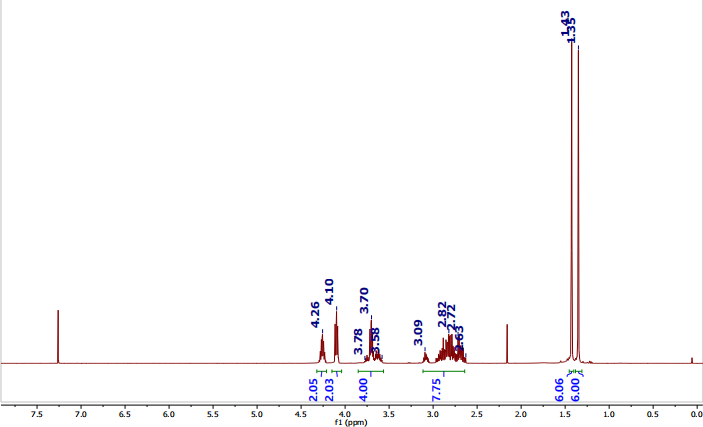


**Fig. S13** 1 H NMR (500 MHz, CDCl3) of compound **1aꞌ**

# Supplementary References

[S1] K. L. Killops, L. M. Campos, C. J. Hawker, Robust, Efficient, and Orthogonal Synthesis of Dendrimers via Thiol-ene “Click” Chemistry. J. Am. Chem. Soc. **130**, 5062-5064 (2008). <https://doi.org/10.1021/ja8006325>

[S2] M. S. Chowdhury, W. Zheng, S. Kumari, J. Heyman, X. Zhang et al., Dendronized fluorosurfactant for highly stable water-in-fluorinated oil emulsions with minimal inter-droplet transfer of small molecules. Nat. Commun. **10**, 4546 (2019). <https://doi.org/10.1038/s41467-019-12462-5>

[S3] S. Shahsavari, C. McNamara, M. Sylvester, E. Bromley, S. Joslin et al., An amine protecting group deprotectable under nearly neutral oxidative conditions.

Beilstein J. Org. Chem. **14**, 1750-1757 (2018). https://doi: 10.3762/bjoc.14.149

[S4] J. M. Sarapas, G. N. Tew, Thiol‐Ene Step‐Growth as a Versatile Route to Functional Polymers. Angew. Chem. Int. Ed. **55**, 15860-15863 (2016). <https://doi.org/10.1002/anie.201609023>

[S5] J. P. E. Human, J. A. Mills, Action of Thionyl Chloride on Carboxylic Acids in Presence of Pyridine. Nature **158**, 877 (1946). <https://doi.org/10.1038/158877a0>

[S6] N. Boehnke, C. Cam, E. Bat, T. Segura, H. D. Maynard, Imine hydrogels with tunable degradability for tissue engineering. Biomacromolecules **16**, 2101-2108 (2015). <https://doi.org/10.1021/acs.biomac.5b00519>

[S7] K. Yusa, L. Zhou, M. A. Li, A. Bradley, N. L. Craig, A hyperactive piggyBac transposase for mammalian applications. Proc. Natl. Acad. Sci. USA **108**, 1531- 1536 (2010). <https://doi.org/10.1073/pnas.1008322108>

[S8] L. N. Randolph, X. Bao, C. Zhou, X. Lian, An all-in-one, Tet-On 3G inducible PiggyBac system for human pluripotent stem cells and derivatives. An all-in- one, Sci. Rep. **7**, 1549 (2017). <https://doi.org/10.1038/s41598-017-01684-6>
